# Decreased sensitivity to aspirin is associated with altered polyamine metabolism in human prostate cancer cells

**DOI:** 10.1007/s00726-015-2143-6

**Published:** 2015-12-24

**Authors:** Jun Li, Gary A. Cameron, Heather M. Wallace

**Affiliations:** Division of Applied Medicine, School of Medicine and Dentistry and School of Medical Sciences, University of Aberdeen, Aberdeen, UK; Cancer Research Centre, Western General Hospital, University of Edinburgh, Edinburgh, UK

**Keywords:** Polyamines, SSAT, Prostate cancer

## Abstract

Aspirin is a well-known analgesic, anti-inflammatory and antipyretic drug and is recognised as a chemopreventative agent in cardiovascular disease and, more recently, in colorectal cancer. Although several studies indicate that aspirin is capable of reducing the risk of developing cancers, there is a lack of convincing evidence that aspirin can prevent prostate cancer in man. In this study, aspirin was shown to be an effective inhibitor of the growth of human prostate cancer cells. In order to investigate the link between polyamine catabolism and the effects of aspirin we used a “Tet off” system that induced the activity of spermidine/spermine *N*^1^-acetyltransferase (SSAT) in human prostate cancer cells (LNCap). Treatment with aspirin was found to decrease induced SSAT activity in these cells. A negative correlation was observed between increased polyamine catabolism via increased SSAT activity and the sensitivity to aspirin. In the presence of increased SSAT activity high amounts of *N*^1^-acetylspermidine and putrescine were observed. These cells were also found to grow more slowly than the non-induced cells. The results indicate that SSAT and its related polyamine metabolism may play a key role in sensitivity of cancer cells to aspirin and possibly other NSAIDs and this may have implications for the development of novel chemopreventative agents.

## Introduction

Prostate cancer is the second most frequently diagnosed cancer in men and the fifth most common cancer overall worldwide (Globocan 2008). Advancing age, race, geographical distribution, diet and family history are the important risk factors contributing to the incidence of this disease (Isaacs et al. 2002). Prostate cancer is mainly a disease of ageing as most cases occur above the age of 60, indicating a slow development of the disease over the years (Khandrika et al. 2009). Men with non-clinical defined prostate cancer at the age of 80 or 90 would be likely to die of other causes rather than the disease itself (Lin and Nelson 2003). Therefore, a strategy of chemoprevention to prevent or delay development of the disease in younger men would be useful and could reduce the prevalence of clinically manifest prostate cancer. LNCaP is an androgen-dependent prostate carcinoma cell line that is considered to represent early stage of prostate cancer development and mimics the disease progression (Dozmorov et al. 2009). Thus, this cell line model is more representative than the other prostate cancer cell lines such as PC-3 and DU-145 to study the effect of aspirin in cancer chemoprevention.

Aspirin use is known to be associated with the chemoprevention of colorectal cancer. However, there is a lack of convincing clinical association between aspirin use and a reduction of prostate cancer incidence, although some studies imply that aspirin use might be inversely related to the risks of developing prostate cancer (Mahmud et al. 2004; Rothwell et al. 2011; Bosetti et al. 2009). In vitro studies reported that NSAIDs could inhibit proliferation of prostate cancer cells such that a selective COX-2 inhibitor, NS398 or celecoxib, and can result in apoptosis (Liu et al. 1998; Hsu et al. 2000). The underlying mechanisms are thought to be related to the inhibition of cyclooxygenases (COX-1 and -2) and decreased production of prostaglandins (Michael et al. 2012). However, it has been suggested that mechanisms that are not dependent on COX inhibition such as inhibition of NF-κB (Babbar et al. 2006) and induction of polyamine catabolism (Hughes et al. 2012) may also be involved in this process.

Polyamines (putrescine, spermidine and spermine) are small polycations present in all mammalian cells. They are indispensable growth factors stimulating cell proliferation through binding to DNA, RNA and proteins (Wallace 2000; Wallace et al. 2003; Criss 2003). This has made the polyamine pathway an attractive target for growth inhibition and for the development of potential novel anticancer strategies. Spermidine/spermine *N*^1^-acetyltransferase (SSAT) is the first enzyme in polyamine catabolism. It acetylates spermidine and spermine to form *N*^1^-acetylspermidine and *N*^1^-acetylspermine, respectively. Acetylation by SSAT is a prerequisite for the export of intracellular polyamines when excess polyamines are present (Shappell et al. 1993). The majority of *N*^1^-acetylated polyamines produced by SSAT are exported out of the cell via the polyamine export system, thus maintaining intracellular polyamine concentrations within predefined limits to maintain optimal rates of cell growth (Pegg 2009).

The SSAT gene is highly inducible by many stimuli including cell stress, toxins, anticancer drugs and polyamine analogues, e.g., *N*^1^,*N*^11^-diethylnorspermine (Mandal et al. 2013). An increase in SSAT activity can lead to an inhibition of cell growth in a number of tumour cell lines (Mandal et al. 2013; Vujcic et al. 2000; Kramer et al. 2008). This is mainly thought to be as a result of a disruption to the cellular polyamine metabolism. Aspirin, as a growth inhibitor in cancer cells, is recognised as an inducer of SSAT, which is associated with the binding of NF-κB on the *Sat1* gene (Babbar et al. 2006).

The aim of this study was to investigate whether there is an association between SSAT activity, the subsequently altered polyamine metabolism and the sensitivity of prostate cancer cells to aspirin. Our hypothesis was that increased SSAT activity will enhance the growth inhibitory effects of the drugs.

## Materials and methods

### Chemicals and labware

Aspirin, acetyl-coenzyme A, aminoguanidine hemi-sulphate, bovine serum albumin, tetracycline hydrochloride, trypan blue, dansyl chloride, 3-(4,5-dimethylthiazolyl)-2,5-diphenyl-tetrazolium bromide (MTT) and 1,7-diaminoheptane were purchased from Sigma-Aldrich, Co. (Poole, UK). Copper sulphate and toluene were purchased from Sigma-Aldrich, Co. (Gillingham, UK). Folin’s-Ciocalteau reagent was purchased from BDH Chemical Co. (Poole, UK). Dithiothreitol was purchased from Sigma Chemical Co. (St. Louis, USA). Geneticin (G418) hygromycin B, RPMI1640 medium, trypsin, 60 mm diameter cell culture dishes and T75 cm^2^ cell culture flasks were purchased from PAA Laboratories Ltd. (Yeovil, UK). Perchloric acid and methanol were purchased from VWR International Ltd. (Lutterworth, UK). Potassium sodium tartrate was purchased from Sigma-Aldrich Co. (Spain). Scintillation cocktail was purchased from GE Healthcare (Buckinghamshire, UK). Tris base was purchased from Melford Laboratories Ltd. (Ipswich, UK). Acetone was purchased from Fisher Scientific Ltd. (Loughborough, UK). l-Proline was purchased from Sigma-Aldrich Co. (China). *N*^1^-Acetylspermidine hydrochloride and *N*^1^-acetylspermine trihydrochloride were purchased from Fluka (Switzerland). Sodium carbonate decahydrate was purchased from Sigma-Aldrich, Co. (Germany). Foetal bovine serum (tetracycline free) was purchased from Labtech International Ltd. (Uckfield, UK). [^3^H] acetyl-coenzyme A and [^14^C] l-ornithine were purchased from PerkinElmer (Cambridge, UK).

### Cell culture

The SSAT human cDNA transfected LNCaP cell line was a kind gift from Drs Carl Porter and Debora Kramer (Roswell Park Memorial Institute, Buffalo, USA). This cell line was genetically transfected with SSAT human cDNA using the tetracycline-off (Tet-off) Advanced Inducible Gene Expression System. Cells were cultured routinely in the presence of tetracycline (Tet) that inhibited the exogenous SSAT expression, producing basal SSAT enzymatic activity (2.13 ± 0.51 pmol/min/mg protein) represented as SSAT^−^ cells. In the absence of Tet, the *SAT1* gene transcription was overexpressed leading to a significant increase in SSAT enzymatic activity (52.38 ± 3.88 pmol/min/mg protein) represented as SSAT^+^ cells. Culture medium: RPMI1640 with l-glutamine + 50 mg/ml G418 + 150 µg/ml hygromycin B + 1 mM aminoguanidine + 10 % (v/v) Tet free Foetal Bovine Serum + 0.4 µg/ml Tet (Tet-free culture medium was the same but without Tet supplementation). The medium was replaced every two days due to a short half-life of Tet in culture (<48 h). LNCap cell seeding density was 2.4 × 10^4^/cm^2^. The cells were cultured routinely in a T75 cm^2^ culture flask containing 15 ml culture medium at 37 °C in a humidified incubator supplemented with 5 % CO_2_.

### Trypan blue cell exclusion assay

The cells were plated in a 60 mm diameter cell culture dish in duplicate and incubated for 48 h prior to treatment with aspirin (2 M stock in ethanol). The cells were trypsinized and stained with trypan blue, and viable cell numbers were counted under a Zeiss light microscope.

### SSAT enzyme activity assay

The method of measuring SSAT activity was described by Wallace and Evans (1998). Cells were lysed in 0.5 ml hypotonic lysis buffer [10 mM Tris (pH 7.2 at 4 °C), 1 mM EDTA and 2.5 mM dithiothreitol]. 40 µl of the cell lysate was used for protein quantification (Lowry assay). The remaining cell homogenate was ultra-centrifuged at 100,000 gav for 70 min at 4 °C to separate cytosol. 60 μl of the cytosol was assayed in duplicate in addition of 10 μl of 30 mM spermidine and 10 μl of 1 M Tris HCl (pH 7.8 at 37 °C), and incubated at 37 °C for 2 min. The reaction was started in ten second cycles on the addition of 10 μl of 250 μM acetyl CoA and 10 μl of 0.33 µCi of [^3^H]-acetyl CoA. All the samples were incubated for 10 min at 37 °C, and then 20 μl of 1 M hydroxylamine was assayed stop the reaction. Samples were boiled for 3 min to precipitate any remaining protein, and centrifuged at 1600*g* for 3 min to pellet the protein. 30 μl of the supernatant was spotted in duplicate onto a Whatman P81 cellulose phosphate-loaded disc. The discs were dried and washed once in tap water, three times in distilled water for 2 min, and finally once in 100 % ethanol to remove unbound [^3^H]-acetyl CoA. The discs were dried again. Each dried paper disc was transferred into a scintillation vial with an addition of 4 ml biodegradable scintillation fluid. The radioactivity of the samples was determined via a tritium protocol by using a Packard 1900 CA Tris carb scintillation analyser. Results were shown as pmol *N*^1^-acetylspermidine formed/min/mg protein.

### ODC enzyme activity assay

ODC activity can be determined by measuring the amount of radiolabelled carbon dioxide ([^14^]CO_2_) produced during the decarboxylation of l-[l-^14^C] ornithine to form putrescine (Tabib 1998). The reaction was carried out in a sealed glass tube and CO_2_ was trapped in benzethonium hydroxide solution. The cell pellet was lysed in 0.5 ml homogenising buffer (10 mM Tris–HCl, 2.5 mM dithiothreitol and 0.1 mM EDTA). 40 µl of the homogenate was taken for protein content determination (Lowry assay). The cell homogenate was centrifuged at a speed of 35,000*g* at 4 °C for 30 min. A stock assay mixes in which 50 µl aliquot was dispensed into each reaction tube. The mixture was composed of 12.5 µl of 1 M Tris–HCl (pH 7.5 at 37 °C), 5 µl of 2 mM pyridoxal 5′-phosphate, 2.5 µl of 250 mM dithiothreitol, 5 µl of 20 mM l-ornithine, and 2.5 µl of l-[l-^14^C] ornithine, and 22.5 µl of deionised water. To each pre-chilled tube, 100 µl of the homogenising buffer and 50 µl aliquot of the assay mixture stock were added. 100 µl benzethonium hydroxide was placed in each well which had been inserted into the rubber stopper for absorbing released [^14^]CO_2_. Finally, 100 µl of the enzyme extract was added to the reaction tube, making a final volume of 250 µl. 100 µl of the homogenising buffer instead of the enzyme extract was used as the blanks. All tubes were sealed tightly with the rubber stoppers with inserted wells and incubated in a shaking water bath for 1 h at 37 °C. 0.3 ml of 2 M perchloric acid was assayed per tube to stop the reaction and the tubes were further incubated for 45 min. All the wells from the tubes were transferred into scintillation vials in addition of 4 ml scintillation fluid. The vials were left in dark overnight. Each sample was counted as disintegrations per minute (dpm) for 10 min using a [^14^C] protocol by a Canberra Packard 1900A liquid scintillation analyser. Results were calculated as pmol ^14^CO_2_ generated/h/mg protein.

### Measurement of intracellular polyamine content by LC–MS–MS

Perchloric acid-extracted intracellular polyamines were quantified using a Thermo Surveyor-TSQ Quantum system (Thermo Scientific, Hemel Hempstead, UK) following derivatization with dansyl chloride in a total run time of 6 min. The derivatised polyamines were resolved using a 5 µl Hichrom HIRPB column (150 × 2.1 mm) and guard cartridge (Hichrom Ltd. Reading, UK) with a mobile phase consisting of water/methanol/formic acid (10/90/0.02). The flow rate was 200 µl/min, the column was maintained at 40 °C and the autosampler at 4 °C with 2 µl injected onto the chromatograph. The column eluent was diverted to waste for the first 2 min of the run. The TSQ Quantum was equipped with an electrospray (ESI) ionisation source and operated in positive ion mode using the following ionisation conditions with a peak width of 0.7 for both Q1 and Q3: spray voltage 4000 V, sheath gas (nitrogen) 30 (arbitrary units), auxiliary gas 0 (arbitrary units), capillary temperature 375 °C and skimmer offset -12 V. Detection was performed in single reaction monitoring (SRM) scan mode with the following SRM transitions and collision energies at a collision gas (Argon) pressure of 1.5 mTorr: putrescine 555.2–170@31 V, 1,7-diaminoheptane (internal standard) 597.2–170@37 V, *N*^1^-acetylspermidine 654.2–100@26 V, *N*^8^-acetylspermidine 654.2–362@26 V, *N*^1^,*N*^12^-diacetylspermine 753.2–100@31 V, spermidine 845.2–360@34 V, *N*^1^-acetylspermine 944.5–100@31 V and spermine 1135.3–360@45 V. The resolution setting for both Q1 and Q3 was 0.2 and each SRM scan time was 0.1 s. Thermo Xcalibur software (v. 2.0.7 SP1) was used for peak integration and quantification was achieved by comparing the peak area ratios with those from calibration curves constructed from the pure standards in the concentration range 0.016–1.0 nmol.

### Total cellular protein determination (Lowry assay)

Total cellular protein content was determined by a modified method from Lowry et al. (1951) using a 96-well plate. A standard linear curve was prepared in the range of 0–250 µg/ml using 0.5 mg/ml bovine serum albumin (BSA) and 0.3 M NaOH. Samples were exposed to 0.13 M Folin–Ciocalteau reagent for 20 min in the dark and absorbance was measured at a wavelength of 690 nm. Total cellular protein content was expressed as mg/culture.

### Polyamine export measurement

Polyamine efflux can be measured by quantifying total [^3^H] labelled polyamines in the extracellular media after prelabelling cells with [^3^H] putrescine (Wallace and Mackarel 1998). The cells were incubated in the addition of 18.5 kBq/ml of [^3^H]-putrescine for 36 h. After replacing with fresh medium and further 12 h incubation, the cells were harvested in a time course. The medium volume was recorded and centrifuged. 1 ml of the medium supernatant was mixed with 0.25 ml of 1 M HClO_4_, and the cell pellet was re-suspended in 1 ml of 0.2 M HClO_4_ for polyamine extraction. 50 µl of the supernatant from the cell extract plus 450 µl of RPMI 1640 medium, and 450 µl from the [^3^H] medium extract plus 50 µl of 0.2 M HClO_4_ were analysed by a Canberra Packard 1900A liquid scintillation analyser. The total radioactivity [disintegrations per minute (dpm)] was the sum of the cell (intracellular) and the medium (extracellular) fraction radioactivity. The extracellular radioactivity was expressed as a percentage of the total radioactivity.

### Statistical method

Statistical analysis was performed using GraphPad software Prism 5 (GraphPad Software, Inc., 2236 Avenida de la Playa, La Jolla, CA 92037, USA). Results were analysed by two-way analysis of variance (ANOVA) with Bonferroni post-tests. A *p* value of less than 0.05 was considered as statistically significant. There are no ethical issues with this manuscript as all work was carried out on cell lines.

## Results

**Fig. 1:**
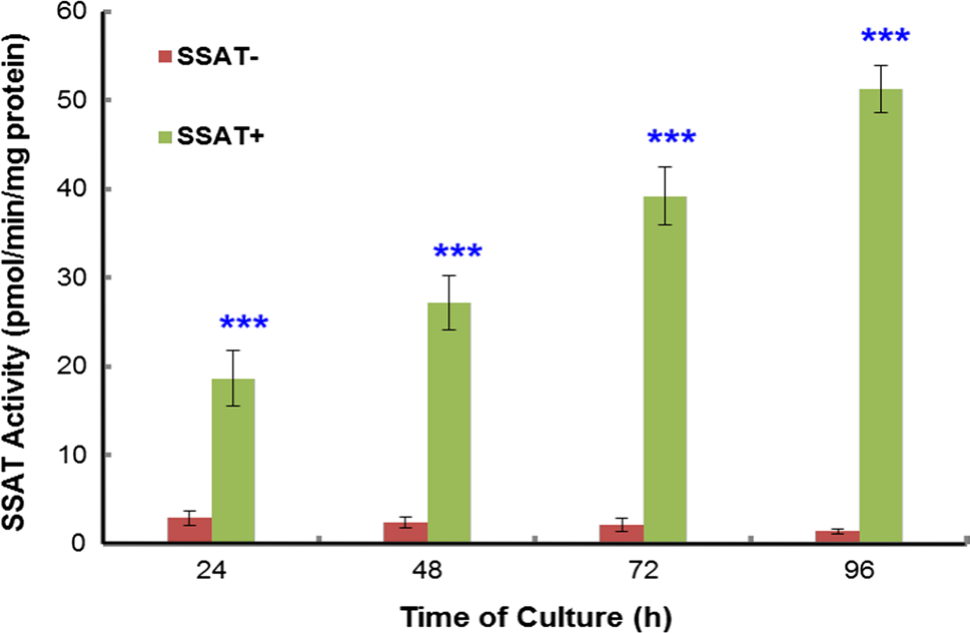
SSAT activity in induced and non-induced LNG53 cells. Cells were seeded at a density of 2.4 × 10^4^/cm^2^ in 60 mm cell culture dish in duplicate. Cells were harvested at 24 h intervals. Media were replaced with fresh media at 24, 72 h. Results shown are mean ± SEM (*n* = 3, with 4 replicate per experiment). Statistical analysis was performed by two-way ANOVA with Bonferroni post-test. ***p < 0.001

**Fig. 2:**
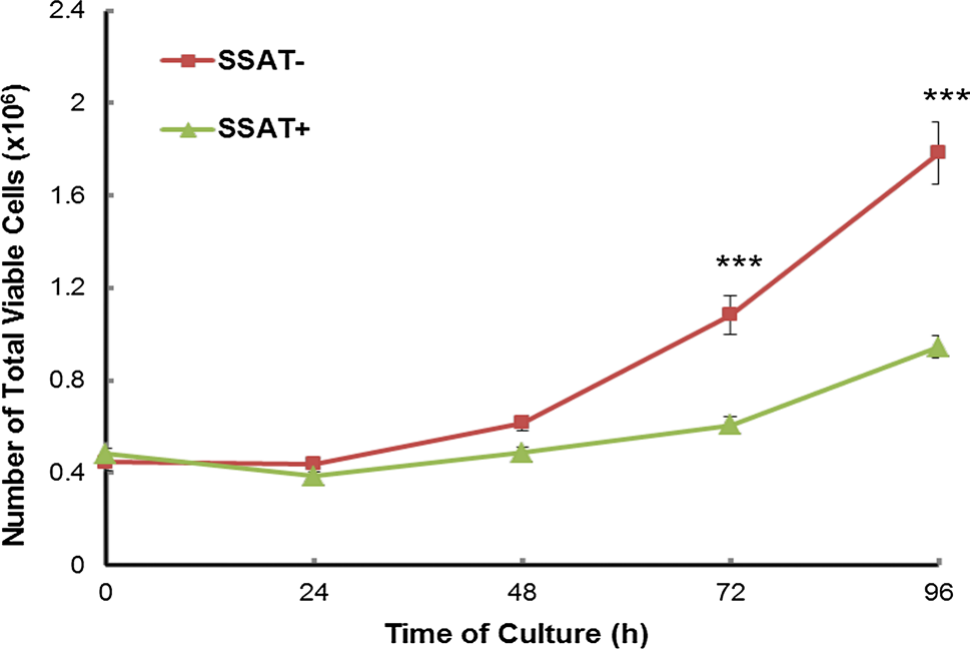
Growth of induced (SSAT^+^) and non-induced (SSAT^−^) cells. Cells were seeded at a density of 2.4 × 10^4^/cm^2^ in 60 mm cell culture dish in duplicate. Cells were harvested at 24 h intervals and viable cell numbers counted using Trypan blue exclusion assay. Results shown are mean ± SEM (*n* = 4–16, with 2 replicate per experiment). Statistical analysis was performed by two-way ANOVA using Prism 5. ***p < 0.001

Initial studies investigated the efficacy of the “Tet-off” system and determined SSAT enzyme activity in both SSAT^**−**^ and SSAT^**+**^ cells over time. Activity in SSAT^**+**^ cells was about 10-18 fold higher than that in SSAT^**−**^ cells and increased in a time-dependent manner (Fig. 1). SSAT activity in SSAT^**−**^ cells remained low throughout (Fig. 1). Thereby, confirming that SSAT activity can be altered by using the “Tet-off” system. Western blot and qPCR confirmed increased protein and mRNA in the SSAT^**+**^ cells (results not shown). The activity in the SSAT^−^ cells was lower than the equivalent wild type LNCaP cells (results not shown). Increased SSAT expression altered the growth of the cells with increased SSAT activity inhibiting cell growth (Fig. 2) and prolonging the generation time (Table 1). The activity of the main polyamine biosynthetic enzyme, ornithine decarboxylase (ODC), was also increased dramatically in SSAT^**+**^ cells (Table 2A).

**Table 1:** Generation time in SSAT^**−**^ and SSAT^**+**^ cells

Time of culture	48 h	72 h	96 h
Cell type	Generation time (h)		
SSAT^−^	39	34	34
SSAT^+^	50	62	51

Cells were seeded at a density of 2.4 × 10^4^/cm^2^ in 60 mm cell culture dish in duplicate. Viable cell numbers were counted using Trypan blue exclusion assay. Mean of the viable cell numbers were used to calculate generation time (Gt). Results were calculated by the equation Gt = log2 × Δt/log(*N*/*N*_0_) related to the total viable cell numbers at 24 h. *Δt* change in time (h), *N* final cell number, *N*_0_ initial cell number

**Table 2:** ODC enzyme activity in SSAT^**−**^ and SSAT^**+**^ cells

Time in culture (h)	24	48	72	96
(A)				
SSAT^−^	1.56 ± 0.38	1.58 ± 0.23	1.30 ± 0.29	1.27 ± 0.20
SSAT^+^	3.89 ± 0.47***	5.73 ± 0.65***	3.35 ± 0.20***	3.48 ± 0.38***

Time of treatment (h)	SSAT^−^		SSAT^+^	
	Control	Aspirin (2 mM)	Control	Aspirin (2 mM)
(B)				
24	0.96 ± 0.13	0.18 ± 0.08***	2.74 ± 0.28	1.70 ± 0.25**
48	0.63 ± 0.14	0.39 ± 0.14	1.16 ± 0.15	0.49 ± 0.10

Cells were seeded at a density of 2.4 × 10^4^/cm^2^ in a 60 mm cell culture dish in duplicate. (A) The cells were harvested at a 24 h interval till 96 h in culture. (B) After 48 h incubation, the cells were treated with 2 mM aspirin for 24 and 48 h. Results shown are mean ± SEM (*n* = 3, with 2 replicate per experiment). Statistical analysis was performed by 2-way ANOVA with Bonferroni post-tests using Prism 5. ** *p* < 0.01, *** *p* < 0.001

Analysis of the intracellular polyamine concentrations in response to altered SSAT activity showed marked increases in *N*^1^-acetylpolyamines and putrescine in SSAT^**+**^ cells (53-, 16-, and fivefold increases in *N*^1^-acetylspermidine, *N*^1^-acetylspermine, and putrescine respectively). Spermine decreased by 57 % but there was little change in spermidine in SSAT^**+**^ cells. Overall, there was little difference in the total polyamine content (Table 3). The alterations of intracellular polyamine concentrations were consistent with the altered enzyme activity of SSAT and ODC in these cells.

**Table 3:** Polyamine concentrations in SSAT^**−**^ and SSAT^**+**^ cells at 96 h in culture

	*N* ^1^-ac-spd	*N* ^1^-ac-spm	Put	Spd	Spm	Total
SSAT^−^	0.12 ± 0.03	0.03 ± 0.01	0.27 ± 0.08	3.08 ± 0.47	11.04 ± 0.82	14.54
SSAT^+^	6.34 ± 0.74***	0.50 ± 0.12***	1.24 ± 0.14***	2.06 ± 0.30	4.75 ± 0.66***	14.89

Cells were seeded at a density of 2.4 × 10^4^/cm^2^ on a 60 mm cell culture dish in duplicate, media were replaced every 48 h. After 96 h incubation, polyamine fraction was extracted by perchloric acid and finally quantified by LC–MS-MS. Values are mean ± SEM with *n* = 6, two replicates per experiment. *** *p* < 0.001

Our hypothesis was that the cells with altered SSAT activity would respond differently to aspirin. However, when the apparent IC_50_ of aspirin, a chemopreventative agent, was determined by MTT assay the values were found to be similar (2.83 ± 0.09 and 2.64 ± 0.16 in SSAT^**−**^ and SSAT^**+**^ respectively. 2 mM aspirin was therefore chosen for the remainder of the study.

The effect of aspirin on cell growth was investigated. Increased SSAT activity decreased the sensitivity of the cells to aspirin, at least, initially. In the treated cells, the growth of SSAT^**−**^ cells was inhibited by 25 % (24 h) and 58 % (48 h), however, in SSAT^**+**^ cells the growth inhibition was 2 % (24 h) and 28 % (48 h), respectively (Table 4). This indicates that the cells with higher SSAT activity are resistant to aspirin treatment at an early stage. Aspirin can inhibit cell proliferation by depleting total intracellular polyamines in vitro (Wallace and Hughes 2006). As shown in Table 5, total polyamines were decreased by aspirin, which corresponded to the results of growth inhibition. These differences in growth inhibition were no longer obvious by 72 and 96 h (Table 4). This is because the SSAT activity in SSAT^**+**^ cells was decreased (from more than 40 to less than 10) by aspirin to a level similar to that in SSAT^**−**^ cells at 72 and 96 h. As a result, the growth inhibition between these two cell types was no longer different.

**Table 4:** Effect of aspirin on the growth inhibition of SSAT^**−**^ and SSAT^**+**^ cells

Cells	Growth inhibition (%)			
Time of treatment (h)	24	48	72	96
SSAT^−^	25	58	80	81
SSAT^+^	2	28	73	83

Cells were seeded at a density of 2.4 × 10^4^/cm^2^ in a 60 mm cell culture dish in duplicate. Cells were treated with aspirin after 48 h incubation and then harvested at 24 h intervals and viable cell numbers counted using Trypan blue exclusion assay. Growth inhibition (%) was calculated as a percentage of the number of cells treated with aspirin against the number of cells of control. The growth inhibition of control cells was known as 0 % (*n* = 3–8, with two replicates per experiment)

**Table 5:** Effect of aspirin on total polyamine concentrations

Time of treatment (h)	Total Polyamines (nmol/mg protein)			
	24	48	72	96
SSAT^−^				
Control	17.1 ± 3.0	21.5 ± 3.8	12.6 ± 3.3	12.1 ± 3.3
Aspirin	14.9 ± 4.5	8.9 ± 1.3	8.1 ± 1.5	8.4 ± 2.1
SSAT^+^				
Control	24.0 ± 6.6	34.8 ± 8.5	17.8 ± 5.0	11.1 ± 2.2
Aspirin	17.0 ± 3.8	10.7 ± 2.4	6.8 ± 1.8	7.1 ± 2.2

Cells were seeded at a density of 2.4 × 10^4^/cm^2^ on a 60 mm cell culture dish in duplicate. Media were replaced after the initial 24 h growth and then at a 48 h interval. Cells were treated with aspirin (2 mM) after 48 h incubation and harvested at a 24 h interval; polyamine fraction was extracted by perchloric acid and finally quantified by LC–MS. Statistical analysis was performed using Two-way ANOVA with Bonferroni post-tests comparing with Control. *p* > 0.05

**Fig. 3:**
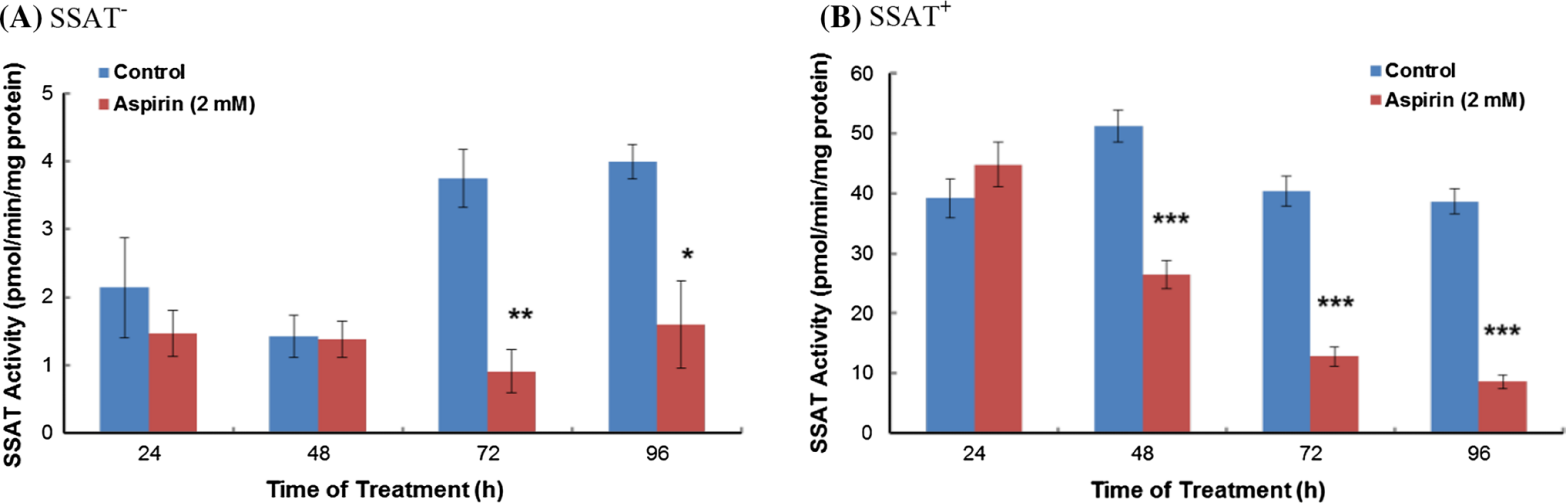
Effect of aspirin on the SSAT enzyme activity. Cells were seeded at a density of 2.4 × 10^4^/cm^2^ on a 60 mm tissue culture dish in duplicate. After 48 h cell growth, the medium was replaced with fresh medium containing aspirin (2 mM). SSAT activity was assayed after 48 h drug exposure. **a** Effect of aspirin on SSAT activity in SSAT^**−**^ cells. **b** Effect of aspirin on SSAT activity in SSAT^**+**^ cells. Values were mean ± SEM (*n* = 3 with 4 replicates per experiment). Statistical analysis was performed using two-way ANOVA with Bonferroni post-tests. *p < 0.05, **p < 0.01, ***p < 0.001

Aspirin at 20 µM was more potent than 100 µM as an inducer of SSAT activity in colon cancer cells (Babbar et al. 2006). This was consistent with our studies in wild type LNCaP prostate cancer cells where aspirin induced SSAT activity (around twofold but not statistically significant) in less than 48 h (results not shown). However, it appeared the higher aspirin concentrations the lower its potency to induce SSAT activity. As aspirin modulates the growth of prostate cancer cells we investigated whether aspirin affected SSAT activity when the enzyme activity was altered. In SSAT^**−**^ cells, the *Sat1* gene expression was blocked by tetracycline, although treatment with aspirin showed a significant decrease of SSAT activity at 72 and 96 h (Fig. 3a). The actual non-induced SSAT enzyme activity in living cells is extremely low (<5 pmol/min/mg protein), this should not indicate a biological consequence. In SSAT^**+**^ cells, treatment with aspirin suppressed the induced activity with a fivefold decrease from 24 to 96 h (Fig. 3b). Aspirin exposure decreased ODC activity in both SSAT^+^ and SSAT^−^ cells (Table 2B). Overall, the results indicate that aspirin can act either as an inducer or an inhibitor to SSAT activity, and it depends on the status of cellular SSAT activity.

**Fig. 4:**
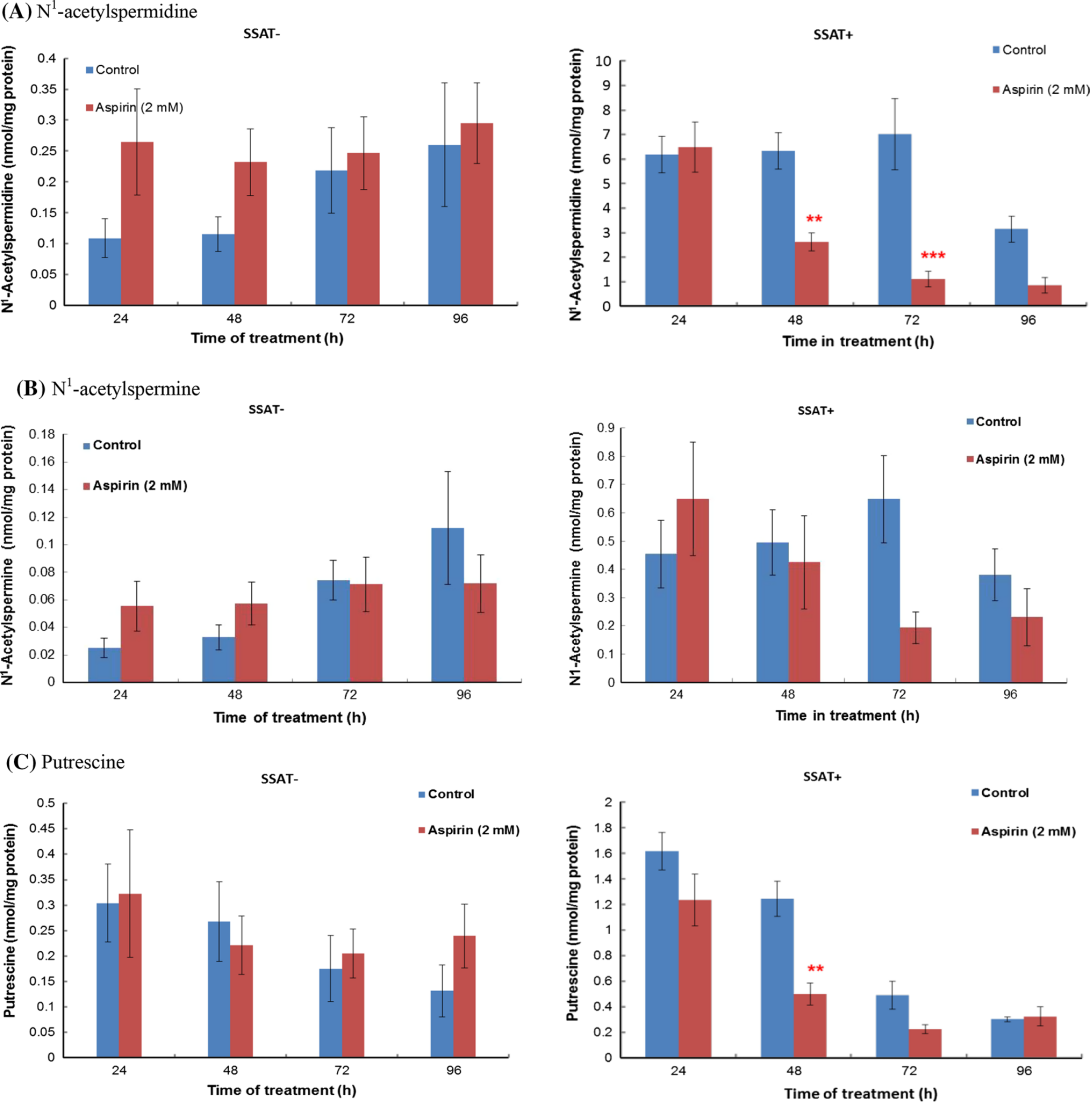

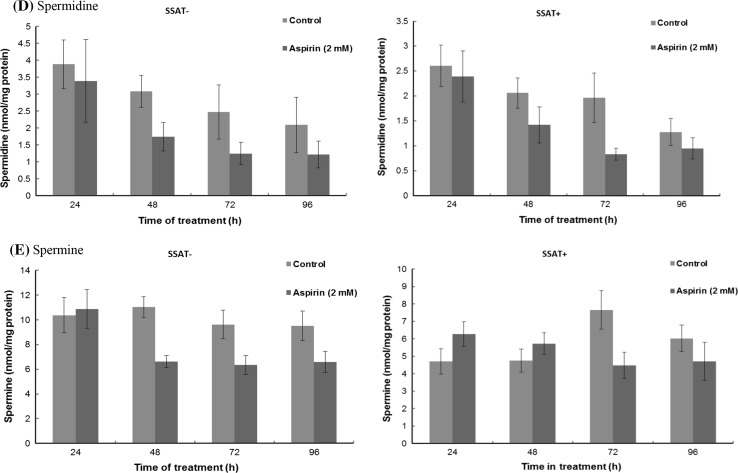
Effect of aspirin on polyamine content in SSAT^**−**^ and SSAT^**+**^ cells. Cells were seeded at a density of 2.4 × 10^4^/cm^2^ on a 60 mm cell culture dish in duplicate. Media were replaced after the initial 24 h growth and then at a 48 h interval. Cells were treated with aspirin (2 mM) after 48 h incubation and harvested at a 24 h interval; polyamine fraction was extracted by perchloric acid and finally quantified by LC–MS-MS. **a** *N* ^1^-acetylspermidine; **b** *N* ^1^-acetylspermine; **c** Putrescine; **d** Spermidine; **e** Spermine; **f** Total polyamines. *Values* are mean ± SEM with *n* = 3–6, two duplicates per experiment). Statistical analysis was performed using Two-way ANOVA with Bonferroni post-tests comparing with Control. *p < 0.05; **p < 0.01; ***p < 0.001

Changes in both ODC and SSAT activities as a result of treatment with aspirin led to alterations in the intracellular concentrations of polyamines. Treatment with aspirin had little effect overall on the intracellular polyamine content of SSAT^−^ cells (Fig. 4a–e). In the SSAT^+^ cells, however, *N*^1^-acetylspermidine and putrescine decreased significantly and *N*^1^-acetylspermine showed a tendency to decrease (Fig. 4a–e). As *N*^1^-acetylspermidine and putrescine are the major polyamines exported from cells when cell growth is inhibited (Wallace et al. 2003) we investigated the effect of aspirin exposure on polyamine export. On the other hand, accumulation of *N*^1^-acetylspermidine and putrescine could render the cells resistant to aspirin within 48 h before majority of them were exported out of the cells.

**Fig. 5:**
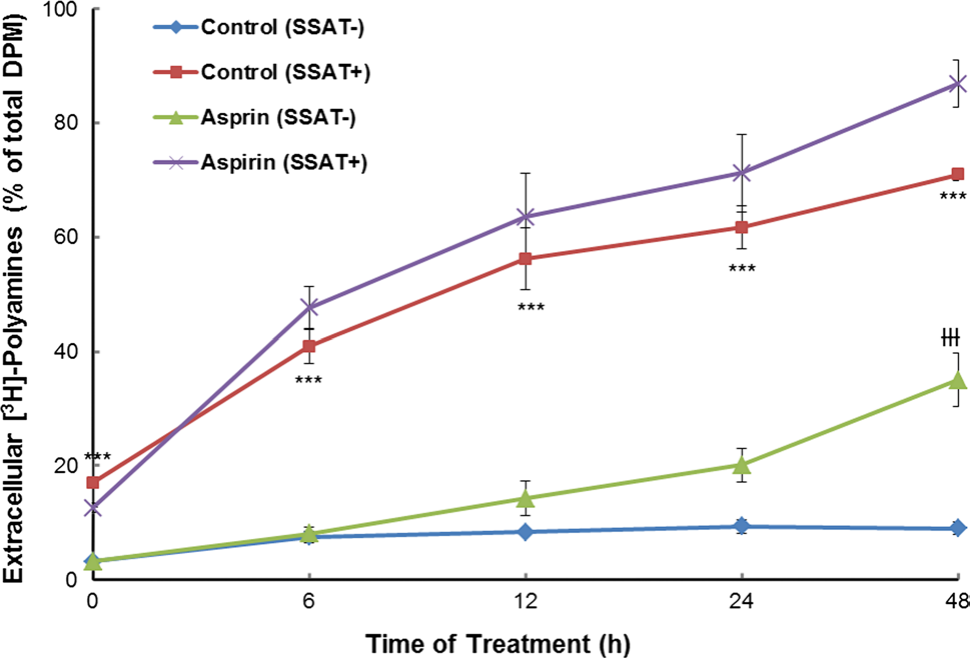
Effect of aspirin on polyamine export. Cells were seeded at a density of 2.4 × 10^4^/cm^2^ in a 60 mm cell culture dish in duplicate and incubated for 24 h. The cells were then incubated with [^3^H]-putrescine for 36 h. After removal of the [^3^H]-putrescine for 12 h, the cells were dosed with the drug(s) and harvested at 0, 6, 12, 24 and 48 h. Radioactivity was quantified using liquid scintillation analyser. Results shown are mean ± SEM (*n* = 3, with two replicates per experiment). Statistical analysis was performed by two-way ANOVA with Bonferroni post-tests using Prism5. Polyamine export was significantly higher in SSAT^**+**^ than SSAT^**−**^ cells without treatment. Aspirin only increased the polyamine export in SSAT^**−**^ cells at 48 h treatment. ***p < 0.001, ^ƗƗƗ^ *p* < 0.001

Polyamine efflux was increased significantly in SSAT^**+**^ in a time dependent manner in contrast to SSAT^**−**^ cells, indicating that polyamine export or efflux was augmented by SSAT overexpression (Fig. 5). This was not affected by aspirin treatment but aspirin did increase export in SSAT^**−**^ cells at 48 h.

## Discussion

Aspirin has been shown to be a chemopreventative agent in colorectal cancer (Thun et al. 2012) and may be useful in preventing other cancers. One mechanism by which aspirin can prevent colorectal cancer development is through inhibition of COX-2 enzyme (Sostres et al. 2014). However, additional COX-independent pathways have been discovered in a number of other studies (Chan et al. 2007; Hubner et al. 2008; Babbar et al. 2006). In our laboratory, NSAIDs such as naproxen have been shown to induce SSAT and cause cytotoxicity in HCT-115 colorectal cancer cells with no COX activity. This indicates a link between SSAT activity and inhibition of cancer cell growth, i.e. SSAT induction leading to depletion of intracellular polyamine pools and decreased cell growth (Hughes et al. 2012). Similarly, aspirin induced SSAT activity in Caco-2 cells that ultimately led to a decrease in cellular polyamine content. Thus, activation of SSAT by aspirin may play a role in chemoprevention in colon carcinogenesis by decreasing the content of polyamine growth factors (Babbar et al. 2006). In this study, overexpression of SSAT appeared to result in a rise in total polyamine content instead of polyamine depletion in SSAT^**+**^ cells. This was consistent with the study by Kee et al. (2004). These results suggest that depletion of intracellular polyamine pools by SSAT induction can be cell type dependent.

There is a lack of clinical evidence that aspirin can prevent prostate cancer development although some studies indicate that there is an inverse association of aspirin use and the development of prostate cancer (Nelson and Harris 2000). In the present study, induced SSAT activity not only inhibited the cell proliferation but desensitised the cells to aspirin. Thus, it provides in vitro evidence that sensitivity of human prostate cancer cells to NSAIDs (aspirin) is associated with the status of cellular SSAT activity.

SSAT overexpression was not only growth inhibitory, but also resulted in resistance to aspirin. A shift from resistance to sensitivity of the cells was simultaneously concomitant with a decrease of induced SSAT activity in response to aspirin. To our knowledge, this is the first time that SSAT activity was found to be associated with aspirin resistance or sensitivity in human tumour cells in vitro. Thus, the efficacy of aspirin in cancer prevention is likely to be associated with inherent cellular SSAT activity. SSAT mRNA was found increased in cancerous human prostate specimens compared with their benign counterparts. In addition, increased SSAT mRNA was more observed in Gleason score 8 than score 2 in these specimens (Bettuzzi et al. 2000). Thereby, cancer cells with high natural SSAT activity might be expected to be more resistant to NSAIDs than cells with low SSAT activity. Our results indicate that the cell resistance to aspirin may occur at an early stage in the treatment and it would reduce with time until SSAT activity is lowered to normal level.

Aspirin tends to induce to SSAT gene expression and activity only if cellular SSAT activity is at normal or low level. When SSAT activity is pre-induced, aspirin, especially at a higher concentration (2 mM), inhibits the SSAT activity. This suggests that whether aspirin is an inducer or inhibitor of SSAT, depends on status of cellular SSAT.

The SSAT reduction by aspirin causing growth inhibition must lead to alterations of intracellular polyamine concentrations since SSAT is a key regulator in polyamine metabolism. As a result of considerable accumulation of intracellular *N*^1^-acetylpolyamines, mainly *N*^1^-acetylspermidine and putrescine, the total polyamine content in SSAT^**+**^ cells tend to rise. It is thought that the accumulation of these polyamines not only inhibits the cell growth but also renders aspirin resistance the cells. A typical phenotype (hair loss and wrinkled skin) of SSAT transgenic mice is believed to be due to the massive accumulation of putrescine in the skin (Pietilä et al. 1997), thus putrescine accumulation may be toxic. On the other hand, low concentrations of spermidine and spermine may also prevent the cells from proliferating and differentiating, since spermidine and spermine are known to be involved in both processes. Our results further confirmed the speculation that either an accumulation or a depletion of intracellular polyamines could be detrimental to the cell. Furthermore, polyamine export is an important part of the regulation of intracellular polyamine concentrations, and this process is also closely associated with SSAT activity. Thereby, the slow proliferation rate of SSAT^**+**^ cells is likely to be linked to the altered balance in the intracellular polyamine content.

The polyamine metabolic pathway has been recognised as a target for cancer therapy and alternations in polyamine content affect cell proliferation. Based on our findings, aspirin is expected to lower SSAT activity in early prostate tumours, therefore enhancing the sensitivity of the tumour cells to the drug and finally preventing tumour development via changing cellular polyamine content. We demonstrated that SSAT acts as a determinant on the chemopreventative effect of aspirin in prostate tumour cells. This determination is closely associated with concentrations of each of the intracellular polyamines.

In summary, the present study suggests that the mechanisms by which aspirin prevents the growth of cancer cells may involve polyamine metabolism as was found in colon cancer cells and may be independent of COX pathways. Modification of intracellular polyamine concentrations via changes in SSAT activity is associated with the rate of prostate cancer cell proliferation but also with the sensitivity of the cells to aspirin. This is the first time this link has been shown. In the use of aspirin to prevent the growth of prostate cancer cells, intracellular polyamine content may play an important role in determining the sensitivity of the cells and SSAT may be a both a target for aspirin and a key regulator of drug response.

## References

[CR1] Babbar N, Gerner EW, Casero RA (2006). Induction of spermidine/spermine *N*^1^-acetyltransferase (SSAT) by aspirin in caco-2 colon cancer cells. Biochem J.

[CR2] Bettuzzi S, Davalli P, Astancolle S, Carani C, Madeo B, Tampieri A, Corti A. (2000) Tumor progression is accompanied by significant changes in the levels of expression of polyamine metabolism regulatory genes and clusterin (sulfated glycoprotein 2) in human prostate cancer specimens. Cancer Res 60:28–34 **(PubMed ID: 10646846)** 10646846

[CR3] Bosetti C, Gallus S, La Vecchia C (2009). Aspirin and cancer risk: a summary review to 2007. Recent Results Cancer Res.

[CR4] Chan AT, Ogino S, Fuchs CS (2007). Aspirin and the risk of colorectal cancer in relation to the expression of COX-2. N Engl J Med.

[CR5] Criss WE (2003). A review of polyamines and cancer. Turkish J Med Sci.

[CR6] Dozmorov MG, Hurst RE. Culkin DJ, Kropp BP, Frank MB, Osban J, Penning TM, Lin HK (2009) Prostate 69:1077–1090. doi:10.1002/pros.20960 10.1002/pros.20960 PMC2755240 19343732

[CR7] Globocan 2008: Cancer incidence and mortality worldwide. International Agency for Research on Cancer, WHO. Available from: http://www.iarc.fr/en/media-centre/iarcnews/2010/globocan2008.php

[CR8] Hsu AL, Ching TT, Wang DS, Song X, Rangnekar VM, Chen CS (2000). The cyclooxygenase-2 inhibitor celecoxib induces apoptosis by blocking Akt activation in human prostate cancer cells independently of Bcl-2. J Biol Chem.

[CR9] Hubner RA, Muir KR, Liu JF, Logan RFA, Grainge MJ, Houlston RS, Members of the UKCAP Consortium (2008). Ornithine decarboxylase G316A genotype is prognostic for colorectal adenoma recurrence and predicts efficacy of aspirin chemoprevention. Clin Cancer Res.

[CR10] Hughes A, Saunders FR, Wallace HM (2012). Naproxen causes cytotoxicity and induces changes in polyamine metabolism independent of cyclo-oxygenase expression. Toxicol Res.

[CR11] Isaacs W, De Marzo A, Nelson WG (2002). Focus on prostate cancer. Cancer Cell.

[CR12] Kee K, Vujcic S, Merali S, Diegelman P, Kisiel N, Powell CT, Kramer DL, Porter CW (2004). Metabolic and antiproliferative consequences of activated polyamine catabolism in LNCaP prostate carcinoma cells. J Biol Chem.

[CR13] Khandrika L, Kumar B, Koul S, Maroni P, Koul HK (2009). Oxidative stress in prostate cancer. Cancer Lett.

[CR14] Kramer DL, Diegelman P, Jell J, Vujcic S, Merali S, Porter CW (2008). Polyamine acetylation modulates polyamine metabolic flux, a prelude to broader metabolic consequences. J Biol Chem.

[CR15] Lin DW, Nelson PS (2003). The role of cyclooxygenase-2 inhibition for the prevention and treatment of prostate carcinoma. Clin Prost Cancer.

[CR16] Liu XH, Tao S, Kirschenbaum A, Levine AC (1998). N398, a selective cyclooxygenase-2 inhibitor, induces apoptosis and down-regulates bcl-2 expression in LNCaP cells. Cancer Res.

[CR17] Lowry OH, Rosebrough NJ, Farr AL, Randall RJ (1951). Protein measurement with the Folin phenol reagent. J Biol Chem.

[CR18] Mahmud S, Franco E, Aprikian A (2004). Prostate cancer and use of nonsteroidal anti-inflammatory drugs: systematic review and meta-analysis. Br J Cancer.

[CR19] Mandal S, Mandal A, Johansson HE, Orjalo AV, Park MH (2013). Depletion of cellular polyamines, spermidine and spermine, causes a total arrest in translation and growth in mammalian cells. Proc Natl Acad Sci.

[CR20] Michael JT, Eric JJ, Carlo P (2012). The role of aspirin in cancer prevention. Nature reviews: Clinical Oncology.

[CR21] Nelson JE, Harris RE (2000). Inverse association of prostate cancer and non-steroidal anti-inflammatory drugs (NSAIDs): results of a case-control study. Oncol Rep.

[CR22] Pegg AE (2009). Mammalian polyamine metabolism and function. IUBMB Life.

[CR23] Pietilä M, Alhonen L, Halmekytö M, Kanter P, Jänne J, Porter CW (1997). Activation of polyamine catabolism profoundly alters tissue polyamine pools and affects hair growth and female fertility in transgenic mice overexpressing spermidine/spermine *N*^1^-acetyltransferase. J Biol Chem.

[CR24] Rothwell PM, Fowkes FGR, Belch JFF, Ogawa H, Warlow CP, Meade TW (2011). Effect of daily aspirin on long-term risk of death due to cancer: analysis of individual patient data from randomised trials. Lancet.

[CR26] Shappell NW, Fogel-Petrovic MF, Porter CW (1993). Regulation of spermidine/spermine *N*^1^-acetyltransferase by intracellular polyamine pools: evidence for a functional role in polyamine homeostasis. FEBS Lett.

[CR27] Sostres C, Gargallo CJ, Lanas A (2014). Aspirin, cyclooxygenase inhibition and colorectal cancer. World J Gastroin Pharmacol Therap.

[CR28] Tabib A (1998). Determination of ornithine decarboxylase activity using [^3^H]ornithine. Methods Mole Biol Polyam Prot.

[CR29] Thun MJ, Jacobs EJ, Patrono C (2012). The role of aspirin in cancer prevention. Nature Rev Clin Oncol.

[CR30] Vujcic S, Halmekyto M, Diegelman P, Gan G, Kramer DL, Janne J, Porter CW (2000). Effects of conditional overexpression of spermidine/spermine *N*^1^-acetyltransferase on polyamine pool dynamics, cell growth, and sensitivity to polyamine analogs. J Biol Chem.

[CR31] Wallace HM (2000). The physiological role of the polyamines. Eur J Clin Invest.

[CR32] Wallace HM, Evans DM (1998) Measurement of spermidine/spermine *N*^1^-acetyltransferase activity. Methods Mol Biol Polyam Prot 79:59–68. Edited by Morgan DM. Totowa, New Jersey: Humana Press Inc. doi:10.1385/0-89603-448-8:59 10.1385/0-89603-448-8:59 9463818

[CR33] Wallace HM, Hughes A (2006) Protective effect of polyamines on NSAID-induced injury and apoptosis. Polyamine Cell Signaling. Physiology, Pharmacology, and Cancer Research. Edited by Wang JY, Casero Jr RA. 273–274. Totowa, New Jersey: Human press Inc

[CR34] Wallace HM, Mackarel J (1998). Regulation of polyamine acetylation and efflux in human cancer cells. Biochem Soc Trans.

[CR35] Wallace HM, Fraser AV, Hughes A (2003). A perspective of polyamine metabolism. Biochem J.

